# Real-world study on microsatellite instability and mismatch repair deficiency testing patterns among patients with metastatic colorectal cancer in Spain

**DOI:** 10.1007/s12094-023-03309-z

**Published:** 2023-08-31

**Authors:** Rocio Garcia-Carbonero, Beatriz González Astorga, Rosario Vidal Tocino, Débora Contreras Toledo, Carles Pericay, Ana Fernández Montes, Esther Falcó, Marta González Cordero, Juan José Reina Zoilo, Vicente Alonso, Nuria Rodríguez Salas, Mireia Gil-Raga, Cristina Santos, David Páez, Beatriz Anton-Pascual, Fernando Aguilar, Pilar Morales

**Affiliations:** 1 Medical Oncology Department, Hospital Universitario 12 de Octubre, IIS Imas12, UCM, Av Cordoba S/N, 28041 Madrid, Spain; 2 Medical Oncology Department, Hospital Universitario Clínico San Cecilio, Granada, Spain; 3 Medical Oncology Department, Complejo Asistencial Universitario de Salamanca, IBSAL, Salamanca, Spain; 4 Medical Oncology Department, Hospital Universitario Central de Asturias, ISPA, Programa Doctorado en Ciencias de la Salud, Universidad de Oviedo, Oviedo, Spain; 5 Medical Oncology Department, Hospital Universitari Parc Taulí, Sabadell, Spain; 6 Medical Oncology Department, Complexo Hospitalario Universitario de Ourense, Ourense, Spain; 7 Medical Oncology Department, Hospital Son Llatzer, Palma, Spain; 8 Medical Oncology Department, Hospital Universitario de Badajoz, Badajoz, Spain; 9 Medical Oncology Department, Hospital Universitario Virgen Macarena, Sevilla, Spain; 10 Medical Oncology Department, Hospital Universitario Miguel Servet, Zaragoza, Spain; 11 Medical Oncology Department, Hospital Universitario La Paz, Madrid, Spain; 12 Medical Oncology Department, Hospital General Universitario de Valencia, Valencia, Spain; 13 Medical Oncology Department, Institute Català d’Oncologia IDIBELL, L’Hospitalet, Barcelona, Spain; 14 Medical Oncology Department, U705 (CIBERER), Hospital de la Santa Creu i Sant Pau, Barcelona, Spain; 15 Medical Affairs Department, MSD Spain, C/Josefa Valcarcel, 38, 28027 Madrid, Spain

**Keywords:** Metastatic colorectal cancer, Microsatellite instability, Mismatch repair deficiency, Microsatellite stable, Real-world evidence

## Abstract

**Purpose:**

Clinical practice guidelines recommend that all patients with metastatic colorectal cancer (mCRC) should be tested for mismatch repair deficiency (dMMR) or microsatellite instability-high (MSI-H). We aimed to describe the dMMR/MSI-H testing practice in patients with mCRC in Spanish centers.

**Methods:**

Multicenter, observational retrospective study that included patients newly diagnosed with mCRC or who progressed to a metastatic stage from early/localized stages.

**Results:**

Three hundred patients were included in the study from May 2020 through May 2021, with a median age of 68 years, and two hundred twenty-five (75%) had stage IV disease at initial diagnosis; two hundred eighty-four patients received first-line treatment, and dMMR/MSI-H testing was performed in two hundred fifty-one (84%) patients. The results of the dMMR/MSI-H tests were available in 61 (24%) of 251 patients before the diagnosis of metastatic disease and in 191 (81%) of 236 evaluable patients for this outcome before the initiation of first-line treatment. Among the 244 patients who were tested for dMMR/MSI-H with IHC or PCR, 14 (6%) were MMR deficient. The most frequent type of first-line treatment was the combination of chemotherapy and biological agent, that was received by 71% and 50% of patients with MMR proficient and deficient tumors, respectively, followed by chemotherapy alone, received in over 20% of patients in each subgroup. Only 29% of dMMR/MSI-H tumors received first-line immunotherapy.

**Conclusion:**

Our study suggests that a high proportion of patients with mCRC are currently tested for dMMR/MSI-H in tertiary hospitals across Spain. However, there is still room for improvement until universal testing is achieved.

*Trial registration*: Not applicable.

**Supplementary Information:**

The online version contains supplementary material available at 10.1007/s12094-023-03309-z.

## Introduction

Colorectal cancer (CRC) is the third most common cause of cancer after breast (females) and lung cancer (males), and the second leading cause of cancer death [1]. Nevertheless, overall survival in patients with CRC has improved in recent years [2], likely due to increased implementation of screening practices and improved therapeutic strategies, including advances in precision medicine and targeted therapies [2, 3]. Consistent with this, current CRC clinical practice guidelines consider that molecular profiling should always include *KRAS*, *NRAS*, and *BRAF* mutations*,* and assessment of mismatch repair deficiency (dMMR) or microsatellite instability (MSI) [4–6].

Several studies indicate that dMMR/MSI-H prognostic value may differ by clinical stage of disease [7]. MSI-H or dMMR is associated with a better prognosis and plays a negative predictive role for adjuvant fluorouracil-based chemotherapy in patients with early stage resected CRC. On the contrary, dMMR/MSI-H is associated with a poorer outcome in patients with advanced disease and an increased response to immune checkpoint inhibitors [7]. In the proof-of-concept phase 2 trial KEYNOTE-016, pembrolizumab, a programmed death 1 (PD-1) inhibitor, was tested in 41 patients with progressive metastatic carcinomas with or without dMMR [8]. This study demonstrated that mismatch-repair status predicted clinical benefit of immune checkpoint blockade with pembrolizumab in many solid tumors of different primary sites, and lead to the first tumor-agnostic indication by the FDA in 2017. To further validate this preliminary activity, a phase 2 study assessed pembrolizumab in 124 dMMR/MSI-H mCRC patients who had received at least one prior line of standard therapy (KEYNOTE-164). This study reported an objective response rate (ORR) of 33%, responses that were profound and durable with PFS rates of 31–34% at 3 years in this heavily pretreated population [9]. More recently, in a phase 3 study conducted in patients with untreated mCRC who were dMMR/MSI-H (KEYNOTE-177), patients randomized to receive pembrolizumab showed a statistically significant and clinically relevant longer progression-free survival (PFS) than those who received standard chemotherapy with or without targeted therapy (median, 16.5 vs. 8.2 months; hazard ratio [HR], 0.60; *p* = 0.0002) with fewer treatment-related adverse events and a trend toward improved survival (median not reached for pembrolizumab vs. 36.7 months in the chemotherapy arm; HR 0.74, 95% CI 0.53–1.03, *p* = 0.036) [10, 11]. Of note, up to 60% of patients crossed over from chemotherapy to anti-PD-1 or anti-PD-L1 therapy upon disease progression, potentially diluting the effect [11]. This study led to the approval of pembrolizumab as monotherapy in the US and Europe as first-line treatment in this population. Finally, nivolumab, another PD-1 inhibitor, alone or in combination with the CTLA-4 inhibitor ipilimumab, was evaluated in the multicohort phase 2 Checkmate-142 study in pretreated mCRC patients. An ORR of 31% was observed for nivolumab alone [12] and of 69% for the combination of nivolumab with ipilimumab, although dual blockade was also associated with greater toxicity [12, 13]. As a result, nivolumab was approved as a single agent in the US or in combination with ipilimumab in the US and Europe for treating dMMR/MSI-H mCRC patients who have progressed after treatment with fluoropyrimidine, oxaliplatin, and irinotecan.

Similarly, dostarlimab has shown efficacy across a wide range of dMMR solid tumors and was also granted a tumor-agnostic FDA approval for MSI-H solid tumors [14]. More recently, the EMA has extended pembrolizumab indications to dMMR/MSI-H advanced or recurrent pretreated endometrial carcinoma, colorectal, gastric, small intestine, or biliary tract cancer [15].

Consistent with these results, the National Comprehensive Cancer Network (NCCN) guidelines recommend universal dMMR/MSI-H testing in all newly diagnosed patients with CRC [6] and the European Society of Medical Oncology (ESMO) mCRC guidelines [16] indicate that dMMR/MSI-H testing can assist clinicians in genetic counseling and has strong predictive value for the use of immune checkpoint inhibitors (ICIs). However, some studies suggest that in clinical practice, dMMR/MSI-H testing is far from being a universal practice [17–19], and in a substantial proportion of CRC patients, it is performed on "red flag" cases (e.g., the presence of other Lynch-related cancer diagnosis, morphological/histological tumor features) or at the clinician´s request [17]. The aim of this study was to describe the dMMR/MSI-H testing practice in patients with mCRC in Spanish centers.

## Material and methods

This was a multicenter, observational, retrospective study conducted in 14 Spanish tertiary hospitals. The study was approved by the Ethics Committee of the Hospital Universitario 12 de Octubre (Madrid, Spain; Reference # 20/322), which acted as a central Research Ethics Committee with Medicines and was also approved by other ethics committees as required by the procedures of each institution. Written informed consent was obtained from every subject except for those subjects who had died before inclusion in the study. All consecutive patients who met the inclusion criteria were enrolled in the study.

### Study subjects

To be included, patients had to be aged 18 years or older and have been diagnosed with mCRC staged according to the 7th edition of the AJCC cancer staging manual [20], with the diagnosis made within 24 months prior to study entry and at least 6 months before study enrollment. Patients could be newly diagnosed with mCRC or have progressed to a metastatic stage from early/localized stages.

### Study assessments

Once included in the study, data were collected retrospectively from the date of diagnosis of metastatic disease until the date of study entry.

The following information was recorded: (1) demographics and clinical characteristics, Eastern Cooperative Oncology Group performance status (ECOG-PS), tumor grade, site of metastases, tumor markers, autoimmune disease at the time of diagnosis, viral infection history, and comorbidities; (2) dMMR/MSI-H testing practice: whether it was performed, requested before initiating first-line treatment, performed in the context of a clinical trial, and type of testing; (3) other molecular biomarker testing at the time of dMMR/MSI-H testing; and (4) first-line treatment for metastatic disease.

### Statistical analysis

We estimated that a sample size of 300 patients would be necessary to detect a proportion of 5% of patients who were dMMR/MSI-H (i.e., the cutoff we used to define a feature as frequent) with a precision of ± 5%.

All statistical analyses were descriptive in nature. For quantitative outcomes, we used the mean and the standard deviation or the median and the interquartile range; categorical outcomes were described with absolute and relative frequencies and the 95% confidence interval when appropriate.

All analyses were performed using IBM SPSS version 26.

## Results

### Patient disposition and characteristics

**Fig. 1:**
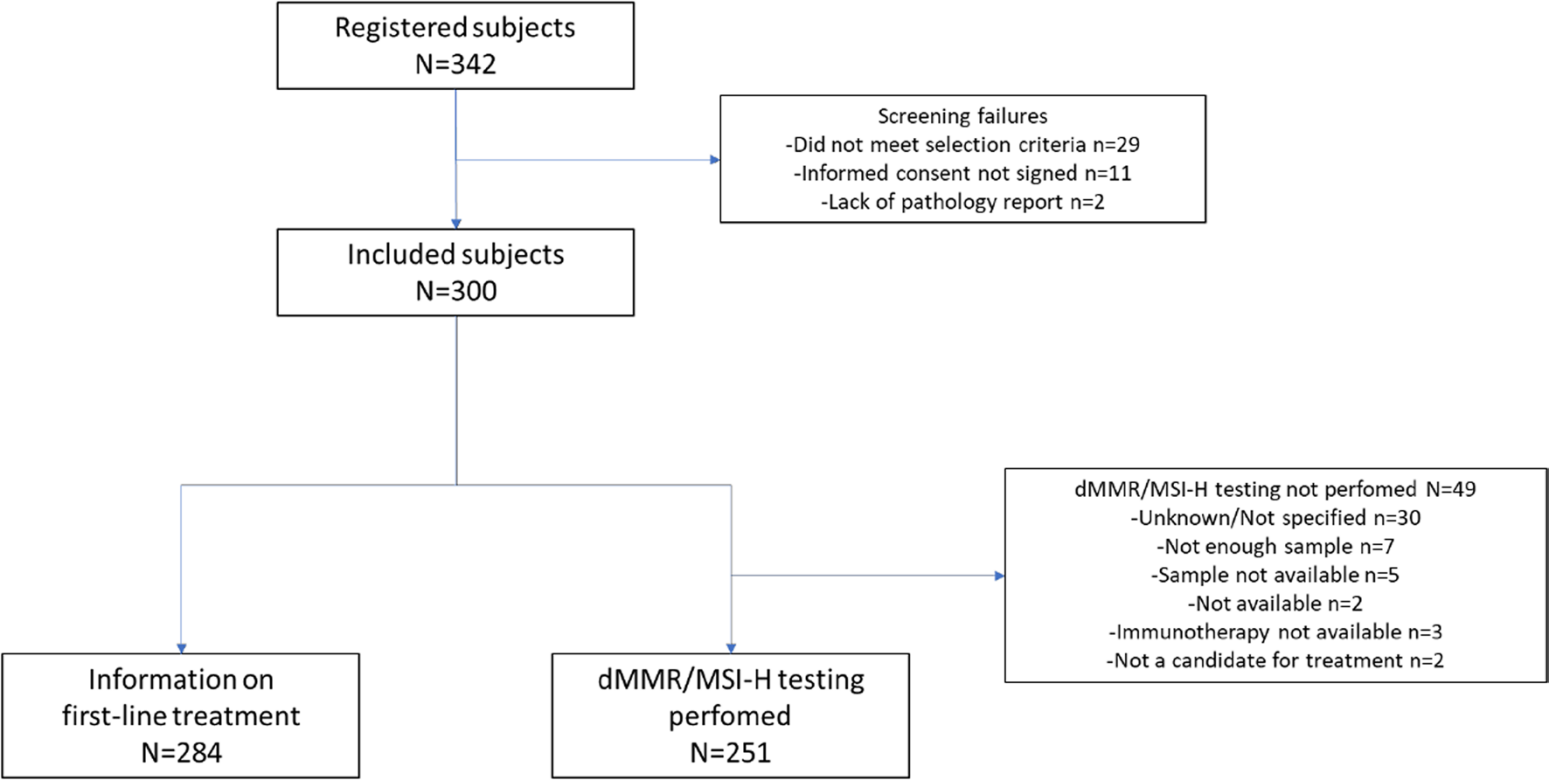
Patient disposition

Between May 25, 2020 and May 14, 2021, a total of 342 patients were screened in 14 Spanish tertiary hospitals from 10 Spanish Autonomous Communities (Cataluña [*n* = 3], Comunidad de Madrid [*n* = 2], Andalucía [*n* = 2], Asturias [*n* = 1], Aragón [*n* = 1], Comunidad Valenciana [*n* = 1], Islas Baleares [*n* = 1], Extremadura [*n* = 1], Galicia [*n* = 1], and Castilla y León [*n* = 1]) (Fig. 1). Three hundred patients were included in the study, out of which two hundred eighty-four received first-line systemic therapy.

The median age of the study population was 68 years, with a slight predominance of males (60%) (Table 1). Most patients (*n* = 225, 75%) had stage IV disease at initial diagnosis, and the most frequent primary tumor locations were the sigmoid colon (*n* = 91, 30%), right colon (*n* = 86, 29%), and rectum (*n* = 56, 19%). At the time of diagnosis of metastatic disease, 240 (80%) showed an ECOG-PS of 0–1, and the most frequent metastatic sites were the liver (*n* = 231, 77%) and lung (*n* = 96, 32%); only 2 patients had brain metastases.

**Table 1:** Demographic and clinical characteristics

Characteristic (*N* = 300)	
Age at diagnosis of metastatic disease, years	
Median (IQR)	68 (60.0–74.0)
> 75 years	60 (20)
Gender (male), *n* (%)	180 (60)
History of autoimmune disease (yes), *n* (%)	13 (4.3)
Stage at initial diagnosis, *n* (%)	
I	4 (1.3)
II	16 (5.4)
III	31 (10.3)
IV	224 (74.7)
Unknown	25 (8.3)
Primary tumor site, *n* (%)	
Right	86 (28.7)
Transverse	19 (6.3)
Left	48 (16.0)
Sigmoid	91 (30.3)
Rectum	56 (18.7)
ECOG-PS at diagnosis of metastatic disease, *n* (%)	
0	108 (36.0)
1	132 (44.0)
2	16 (5.3)
≥ 3	3 (1.0)
Unknown	41 (13.7)
Time since initial diagnosis (months), mean (SD)	18.3 (13.1)
Time since diagnosis of metastatic disease (months), mean (SD)	13.9 (6.2)
Most frequent (≥ 5%) location of metastases, *n* (%)	
Liver	231 (77.0)
Lung	96 (32.0)
Retroperitoneal affected organs	59 (19.7)
Lymph nodes	19 (6.3)

*ECOG-PS* Eastern Cooperative Oncology Group Performance Status, *IQR* interquartile range, *SD* standard deviation

### Characteristics and results of dMMR/MSI-H testing

dMMR/MSI-H testing was performed in 251 (84%) patients (Fig. 1); the test was performed in the participant center for 215 (86%) of these patients, corresponding to 13 out of 14 centers (Table 2). The results of the dMMR/MSI-H testing were available in 24% of the patients before the diagnosis of metastatic disease and in 81% of the evaluable patients for this outcome before the initiation of first-line treatment. There was high variability among different centers regarding the time elapsed between the diagnosis of metastatic disease and the obtention of dMMR/MSI-H results, with a median of 13 days if the test was performed in-house and a median of 78 days if the test was performed at a reference laboratory.

**Table 2:** Characteristics and overall results of dMMR/MSI-H testing

Variable	All patients		dMMR/MSI-H subgroup	
	*N*		*N*	
dMMR/MSI-H testing performed (yes), *n* (%)	300	251 (83.7)		
Institution where the testing was performed, *n* (%)	251		14	
In-house		215 (85.7)		13 (92.9)
Reference lab		32 (12.7)		1 (7.1)
Unknown		4 (1.6)		0 (0.0)
Availability of dMMR/MSI-H testing results (Yes), *n* (%)				
Before mCRC diagnosis	251	61 (24.3)	14	6 (57.1)
Before first-line treatment	236	191 (80.9)	14	11 (78.6)
Time from mCRC diagnosis to dMMR/MSI-H testing results (days), median (IQR)^a^				
Total	246^b^	− 15.0 (− 41.0; 1.0)	14	− 11.5 (− 36.0; 223.0)
In-house	215	− 13.0^c^ (− 31.0; 5.0)	13	− 12.0 (− 36.0; 18.0)
Reference lab	31	− 78.0^c^ (− 115.0; − 35.0)	1	334.0
Methods for dMMR/MSI-H testing, *n* (%)	251^d^			
IHC		157 (62.5)		8 (57.1)
IHC + PCR		27 (10.8)		2 (14.2)
IHC + NGS		23 (9.2)		0 (0)
IHC + PCR + NGS		3 (1.2)		3 (21.4)
PCR		28 (11.2)		1 (7.1)
PCR + NGS		6 (2.4)		0 (0)
NGS		7 (2.8)		0 (0)
dMMR/MSI-H status according to IHC and/or PCR, *n* (%)^e^	244			
pMMR		230 (94.3)		–
dMMR		14 (5.7)		–

*dMMR/MSI-H* mismatch repair deficiency or microsatellite instability-high, *IHC* immunohistochemistry, *IQR* interquartile range, *PCR* polymerase chain reaction, *mCRC* metastatic colorectal cancer; NGS, next-generation sequencing

^a^Only patients with dates for dMMR/MSI-H testing and first-line treatment initiation were evaluable

^b^dMMR/MSI-H test results and collection dates are missing for 22 patients

^c^The negative numbers refer to dMMR/MSI-H testing results before mCRC diagnosis

^d^The results were not available in 13 patients

^e^Includes only patients who were tested with IHC and/or PCR

Immunohistochemistry (IHC) was the most used method to detect dMMR/MSI-H, and it was performed in 210 (84%) out of 251 evaluable patients. The other commonly used methods were polymerase chain reaction (PCR) and next-generation sequencing (NGS), which were used in 64 (26%) and 39 (16%) patients, respectively. It was not common to use more than one method at a time, with IHC plus PCR and IHC plus NGS being used in 11% and 9% of the patients, respectively (Table 2). NGS was used in routine clinical practice in 36 out of 39 patients. Among the 244 patients who were tested for dMMR/MSI-H with IHC or PCR, 14 (6%) were MMR deficient or MSI-H, and among those evaluated with IHC, the most frequent profile was MLH1/PMS2 deficient (9 [4.3%] of 210 patients). The characteristics of patients according to the dMMR/MSI-H status are presented in supplementary table 1.

### Molecular profiling of mCRC

The availability of the standard biomarkers recommended by clinical practice guidelines is presented in Table 3. Among the 284 treated patients, dMMR/MSI-H testing was performed in 240 (85%) patients, *KRAS* testing was performed in 269 (95%) patients, and *NRAS* and *BRAF* testing were performed in 144 (94%) of the *KRAS*-wild type patients.

**Table 3:** Availability of biomarkers recommended by clinical practice guidelines among treated patients

Standard biomarker	All treated patients *N* = 284	KRAS-wild type^a,b^ *N* = 153	KRAS-mutated^a^ *N* = 116
dMMR/MSI-H testing	240 (84.5)	125 (85.6)^b^	99 (85.3)
*KRAS* testing^a^	269 (94.7)	–	–
*NRAS* testing	197 (69.4)	144 (94.1)	53 (45.7)
*BRAF* testing	216 (76.1)	144 (94.1)	72 (62.1)

dMMR/MSI-H, mismatch repair deficiency or microsatellite instability-high

^a^The test was not done in 25 patients

^b^The number of evaluable patients for dMMR/MSI-H testing was 146 with IHC and/or PCR results. In the five patients in whom the determination was made only by NGS, the results are not available

In addition, at least one of the following biomarkers not universally recommended by clinical practice guidelines was determined in 35 (12.3%) of treated patients: ALK, ROS, RET, NTRK, HER2, PIK3CA, EGFR, and MET.

### Pattern of treatment according to dMMR/MSI-H status

Regarding the dMMR/MSI-H status, the most frequent type of first-line treatment received was the combination of chemotherapy and biological therapy in 71% and 50% of the patients who were microsatellite stable and dMMR/MSI-H, respectively, and chemotherapy alone in over 20% of the patients in each subgroup (Table 4). Immunotherapy as monotherapy was prescribed to 3 of the 14 patients who were dMMR/MSI-H (i.e., pembrolizumab *n* = 2, nivolumab/ipilimumab *n* = 1), and in combination with chemotherapy and biological therapy to one of the 14 patients (i.e., irinotecan plus bevacizumab plus dostarlimab).

**Table 4:** First-line treatment received by dMMR/MSI-H status

Treatment type	Microsatellite stable *N* = 230	dMMR/MSI-H *N* = 14
Any, *n* (%)	219 (95.2)	14 (100)
By type of treatment, *n* (%)^a^		
Chemotherapy and biological	156 (71.2)	7 (50.0)
Chemotherapy only	54 (24.7)	3 (21.4)
Chemotherapy, biological, and others	4 (1.8)	–
Biological only	2 (0.9)	–
Biological and others	1 (0.5)	–
Chemotherapy and others	1 (0.5)	–
Immunotherapy only	–	3 (21.4)^b^
Chemotherapy, biological, and immunotherapy	–	1 (7.1)^c^

^a^Percentages were calculated over those who received treatment

^b^Pembrolizumab *n* = 2, nivolumab/ipilimumab *n* = 1

^c^Irinotecan plus bevacizumab plus dostarlimab

## Discussion

Conducting research under real-world practice conditions with a representative sample of mCRC patients from Spain showed that dMMR/MSI-H testing is frequent but not universally established. The most frequent method for testing was IHC, and the prevalence of dMMR/MSI-H was 6%.

Our study found that dMMR/MSI-H testing was performed in 84% of the patients with mCRC and in 85% of those who received treatment. These figures are higher than those reported in other studies from Australia and in the United States [17, 18]. A survey among pathologists from Australia, conducted to assess Lynch syndrome tumor screening practices, reported that from 36 laboratories that received CRC specimens, only 47% reported following a universal testing approach, 30% performed the test only for “red flag” cases, and 6% of tests were performed upon the clinician´s request [17]. Using data from a real-world registry of 23 practices and 258 oncologists in the United States, Gutierrez et al. [18] reported that among 1497 patients with confirmed mCRC, dMMR/MSI-H testing was performed in 51% of patients. The higher figure reported in our study is likely due to the different timeframes in which these studies were conducted. Our study was conducted between May 2020 and May 2021, while the Australian survey was performed in 2015, and the US study included patients diagnosed between January 2013 and December 2017. Interestingly, in the latter study, within that timeframe, dMMR/MSI-H testing increased from 25% in 2013 to 47% in 2015 and 57% in 2017 [18]. Furthermore, in a study that took place in the US in 152,993 adult patients with CRC diagnosed between 2010 and 2012, only 28% of the patients had undergone dMMR/MSI-H testing [19]. The improvement in molecular profiling observed in recent years is also reflected in our study, with a high proportion of patients with KRAS wild type who were tested for NRAS and BRAF (i.e., 94%). In contrast, in a previous study carried out in Spain between November 2016 and April 2017, almost 50% of the patients who were RAS wild type had an unknown BRAF mutation status [21]. As stated previously, according to the current NCCN guidelines for colorectal cancer, dMMR/MSI-H testing is recommended for all newly diagnosed CRC patients [6]. While dMMR/MSI-H testing in our study was frequent, it was not universally performed. Based on its predictive value for immunotherapy in patients with dMMR/MSI-H and after the establishment of pembrolizumab monotherapy as first-line treatment, and pembrolizumab or nivolumab with or without ipilimumab for second-line, several studies have suggested that dMMR/MSI-H testing in patients with mCRC is cost-effective [22, 23].

The most frequent method for dMMR/MSI-H testing was IHC, which was performed in 84% of the patients. This is consistent with the clinical practice guideline recommendations. For instance, the ESMO guidelines recommend with a strong grade of consensus IHC for dMMR/MSI-H testing, that utilizes antibodies against the four main MMR proteins, because it is widely available and feasible to perform in every center, leaving PCR molecular testing for those cases with indeterminate or negative IHC results and ‘red flag’ features [24].

The prevalence of dMMR in patients with CRC is higher in early stages than in advanced disease, and higher in western populations [25, 26]. The prevalence in our study (6%) overlaps with that reported in two recent series in real-world practice in the US using the Veterans Affairs Health Care System [27] and the MD Anderson Cancer Center [28] databases. It is also within the range shown in a recent review that included a pooled analysis [26]. Using data from 14 studies and 8,156 patients, Lorenzini et al. reported a pooled prevalence of MSI-H of 13% (10–16%), and by pooling 4 studies corresponding to 11,434 patients, they found a dMMR pooled prevalence of 10% (5–15%) [26]. When analyzed by stage, the prevalence was 20% (10–32%) for stages I–II (4 studies and 888 patients) and 9% (3–16%) for stages III–IV (4 studies; 873 patients) [26].

The major limitation of our study is that it was conducted in a limited number of tertiary hospitals; however, we consider the sample to be representative of patients in the metastatic setting.

In conclusion, our study suggests that in Spanish tertiary hospitals, a high proportion of patients with mCRC is currently tested for dMMR/MSI-H. However, there is still room for improvement until universal testing is established. Identification of patients with dMMR/MSI-H is essential and should be a health care priority not only for CRC but also for other solid tumors, such as endometrial, gastric, biliary tract or small intestine tumors, in which immunotherapy has been recently approved as a first- or second-line treatment and has shown relevant benefits compared with the current standard of care.

### Supplementary Information

Below is the link to the electronic supplementary material.

Supplementary file1 (DOCX 22 KB)

## Data Availability

The data that support the findings of this study are available from the corresponding author upon reasonable request.
