# Supplementary material for: Real-world study on microsatellite instability and mismatch repair deficiency testing patterns among patients with metastatic colorectal cancer in Spain
Source: Clin Transl Oncol. 2023 Aug 31;26(4):864–71. doi: 10.1007/s12094-023-03309-z (PMC10981578; doi:10.1007/s12094-023-03309-z)
Supplement: Supplementary file 1 — Supplementary file1 (DOCX 22 KB) [file 12094_2023_3309_MOESM1_ESM.docx]

**Supplementary information Supplementary table 1**

**Patient Characteristics by dMMR/MSI-H status**

| **Characteristic (N=300)** | **Microsatellite stable**  **N=230** | **dMMR/MSI-H**  **N=14** |
| --- | --- | --- |
| Age at diagnosis of metastatic  disease (years), median (IQR) | 67.0 (59.0-74.0) | 61.0 (51.0-72.0) |
| Gender (male), n (%) | 137 (59.6) | 7 (50.0) |
| History of autoimmune disease  (yes), n (%) | 9 (3.9) | 1 (7.1) |
| Stage at initial diagnosis, n (%) |  |  |
| I | 4 (1.7) | 0 (0.0) |
| II | 11 (4.8) | 1 (7.1) |
| III | 23 (10.0) | 3 (21.4) |
| IV | 172 (74.8) | 9 (64.3) |
| Unknown | 20 (8.7) | 1 (7.1) |
| Primary tumor site, n (%) |  |  |
| Right | 59 (25.7) | 9 (64.3) |
| Transverse | 13 (5.7) | 3 (21.4) |
| Left | 40 (17.4) | 0 (0.0) |
| Sigmoid | 76 (33.0) | 2 (14.3) |
| Rectum | 42 (18.3) | 0 (0.0) |
| ECOG-PS at diagnosis of  metastatic disease, n (%) |  |  |
| 0 | 83 (36.1) | 5 (35.7) |
| 1 | 101 (43.9) | 8 (57.1) |

| 2 | 9 (3.9) | 1 (7.1) |
| --- | --- | --- |
| ≥3 | 3 (1.3) | 0 (0.0) |
| Unknown | 34 (14.8) | 0 (0.0) |
| Time since initial diagnosis  (months), mean (SD) | 17.6 (11.9) | 27.0 (24.0) |
| Time since diagnosis of metastatic disease (months),  mean (SD) | 13.8 (6.1) | 15.6 (5.9) |
| Most frequent (≥5%) location of  metastases, n (%) |  |  |
| Liver | 179 (77.8) | 8 (57.1) |
| Lung | 76 (33.0) | 4 (28.6) |
| Retroperitoneal affected organs | 41 (17.8) | 4 (28.6) |
| Lymph nodes | 15 (6.5) | 1 (7.1) |
| Biomarkers |  |  |
| BRAF mutated, n/N (%) | 10/167 (6.0) | 3/12 (25.0) |
| KRAS mutated, n/N (%) | 100/216 (46.3) | 4/14 (28.6) |
| NRAS mutated, n/N (%) | 13/152 (8.6) | 0/11 (0.0) |

ECOG-PS, Eastern Cooperative Oncology Group Performance Status; IQR, interquartile range; n, number of patients with the characteristic; N, number of patients evaluated; SD, standard deviation
